# Prospective evaluation of the capillaroscopic skin ulcer risk index in systemic sclerosis patients in clinical practice: a longitudinal, multicentre study

**DOI:** 10.1186/s13075-018-1733-6

**Published:** 2018-10-25

**Authors:** Ulrich A. Walker, Veronika K. Jaeger, Katharina M. Bruppacher, Rucsandra Dobrota, Lionel Arlettaz, Martin Banyai, Jörg Beron, Carlo Chizzolini, Ernst Groechenig, Rüdiger B. Mueller, François Spertini, Peter M. Villiger, Oliver Distler

**Affiliations:** 1 Department of Rheumatology, University Hospital Basel, Petersgraben 4, 4032 Basel, Switzerland; 2 Bellikon, Switzerland; 3 Department of Rheumatology, University Hospital Zurich, Zurich, Switzerland; 4 Institut Central—Hôpital du Valais, Sion, Switzerland; 5 Kantonsspital Luzern, Luzern, Switzerland; 6 Actelion Pharma Schweiz AG, Baden, Switzerland; 7 Immunology & Allergy, University Hospital and School of Medicine, Geneva, Switzerland; 8 Kantonsspital Aarau, Aarau, Switzerland; 9 Kantonsspital St. Gallen, St. Gallen, Switzerland; 10 Division of Immunology and Allergy, Centre Hospitalier Universitaire Vaudois, Lausanne, Switzerland; 11 Department of Rheumatology, Immunology and Allergology, University Hospital and University of Bern, Bern, Switzerland

**Keywords:** Systemic sclerosis, Nailfold capillaroscopy, Capillaroscopic skin ulcer risk index, Inter-rater reliability, Digital ulcer prediction

## Abstract

**Background:**

Nailfold capillaroscopy (NC) is an important tool for the diagnosis of systemic sclerosis (SSc). The capillaroscopic skin ulcer risk index (CSURI) was suggested to identify patients at risk of developing digital ulcers (DUs). This study aims to assess the reliability of the CSURI across assessors, the CSURI change during follow-up and the value of the CSURI in predicting new DUs.

**Methods:**

This multicentre, longitudinal study included SSc patients with a history of DUs. NC images of all eight fingers were obtained at baseline and follow-up and were separately analysed by two trained assessors.

**Results:**

Sixty-one patients were included (median observation time 1.0 year). In about 40% of patients (assessor 1, *n* = 24, 39%; assessor 2, *n* = 26, 43%) no megacapillary was detected in any of the baseline or follow-up images; hence the CSURI could not be calculated.

In those 34 patients in whom CSURI scores were available from both assessors (26% male; median age 57 years) the median baseline CSURI was 5.3 according to assessor 1 (IQR 2.6–16.3), increasing to 5.9 (IQR 1.3–12.0) at follow-up. According to assessor 2, the CSURI diminished from 6.4 (IQR 2.4–12.5) to 5.0 (IQR 1.7–10.0).

The ability of a CSURI ≥ 2.96 category to predict new DUs was low (for both assessors, positive predictive value 38% and negative predictive value 50%) and the inter-assessor agreements for CSURI categories were fair to moderate.

**Conclusions:**

In this study, around 40% of patients could not be evaluated with the CSURI due to the absence of megacapillaries. Clinical decisions based on the CSURI should be made with caution.

**Trial registration:**

Current Controlled Trials, ISRCTN04371709. Registered on 18 March 2011.

**Electronic supplementary material:**

The online version of this article (10.1186/s13075-018-1733-6) contains supplementary material, which is available to authorized users.

## Background

Systemic sclerosis (SSc) is a chronic connective tissue disease characterised by endothelial cell dysfunction and fibrosis of the skin and internal organs [1, 2]. Microangiopathy is one of the main histopathologic features detectable early in the course of the disease [3]. A gradual progression of vascular abnormalities has been observed during SSc progression [4]. Nailfold capillaroscopy (NC) is an imaging technique that detects morphological abnormalities of nailfold microcirculation. Furthermore, NC is an important tool for the classification and diagnosis of SSc in clinical practice [5, 6]. The three NC patterns early, active and late were found to be associated with Raynaud’s phenomenon (RP) as well as with the duration of the disease, possibly reflecting SSc evolution [4]. Although the diagnostic value of the NC patterns is well defined [7], different methodologies have been proposed to assess quantitative NC abnormalities in the follow-up of patients with SSc. However, their clinical applicability remains uncertain.

Sebastiani et al. [8] proposed the capillaroscopic skin ulcer risk index (CSURI) in 2009, as a quantitative measure of nailfold capillary damage that predicts the appearance of new digital ulcers (DUs) as well as the persistence of pre-existing DUs [8, 9]. The CSURI is based on the number of capillaries in the distal nailfold capillary row and the number of megacapillaries, as well as the maximum diameter of the megacapillaries on capillaroscopic evaluation [8, 9].

In order to gain better insight into the value of monitoring quantitative NC abnormalities in clinical practice, this multicentre study was designed to describe the reliability of the CSURI across different trained assessors, to describe the change of CSURI during follow-up, to assess the value of the CSURI in predicting new DUs and to assess associations between the CSURI and demographic and disease characteristics.

## Methods

### Study population and design

This multicentre, prospective, observational study was carried out across eight sites in Switzerland between 2011 and 2015. Adult patients fulfilling the 1980 American College of Rheumatology criteria for SSc and with a history of DUs were included [10]. DUs were defined as a painful area with visually discernible depth and a loss of continuity of epithelial coverage which can be denuded or covered by a scab or necrotic tissue and is vascular in origin. Fissures, paronychia, extrusion of calcium or ulcers over the metacarpophalangeal joints or elbows are not regarded as DUs. In order to be included in this analysis, patients were also required to have at least one follow-up visit; if a patient had more than one follow-up visit, the last one was chosen as the follow-up visit. All inclusion and exclusion criteria are summarised in Additional file 1: Table S1.

This study was approved by the centres’ ethic committees and each patient provided written informed consent.

Demographic patient characteristics and routine clinical data were recorded prospectively on a web-based electronic data capture system. Table 1 presents a description of the data collected. Patients underwent NC at baseline and at follow-up visits. Follow-up visits were performed if deemed necessary by the centres’ physicians, but were recommended at 3, 6 and 12 months. Regular external monitoring with primary data verification was performed to ensure data quality.

**Table 1:** Description of collected data

Demographics	
Age (years)	
Sex (female/male)	
Smoking habit (never smoker/ex-smoker/current smoker)	
Disease characteristics	
Time since RP onset (years)	
Time since first non-RP manifestation (years)	
Cutaneous involvement (limited/diffuse)	
Modified Rodnan skin score (range 0–51)	
Erectile dysfunction (yes/no; defined as a score below 22 in the International Index for Erectile Dysfunction-5 [19])	
Kidney involvement (yes/no; defined as proteinuria)	
History of renal crisis (yes/no)	
RP condition score (range 0–10)	
DUs (yes/no; defined as a painful area with visually discernible depth and a loss of continuity of epithelial coverage, which can be denuded or covered by a scab or necrotic tissue and is vascular in origin; DUs do not include fissures, paronychia, extrusion of calcium or ulcers over the metacarpophalangeal joints or elbows.)	
Time since first DU (years)	
Number of DUs	
Major digital vascular complications (none/soft tissue infection/gangrene/autoamputation)	
Laboratory (measured according to local standards in the respective centres)	
Antinuclear autoantibody positivity (yes/no)	
Anticentromere autoantibody positivity (yes/no)	
Anti-topoisomerase autoantibody positivity (yes/no)	

*DU* digital ulcer, *RP* Raynaud’s phenomenon

Prior to commencing the study, the study sites’ investigators were trained at an investigator meeting to perform NC. The nailfolds of eight fingers (digits 2–5 on both hands) were examined using the same NC device equipped with a 200× lens with LED illumination and an immersion fluid contact adapter (Optilia instruments AB, Sollentuna, Sweden) in all centres. Four images across the nailfold quadrants of each finger were obtained. Digital NC images were stored centrally and examined separately at the end of the study by two identically trained central assessors (UAW and OD). The central assessors were blinded for the patients, the temporal sequence of the fingers and the scoring results of the other assessor. In each NC image, the assessors assessed the total number of capillaries in the distal row, the number of megacapillaries and the maximum diameter of the megacapillaries. Additionally, the images were also evaluated locally at the centres (local assessors). The qualitative assessment—that is, the NC pattern (early/active/late)—was performed by one additional central assessor (RD).

The presence of at least one megacapillary is necessary to calculate the CSURI [8, 9]. The CSURI is calculated for only one image per patient per time point; this image is identified based on the lowest number of capillaries in the distal row as the first criterion and subsequently the highest number of megacapillaries as the second criterion [8, 9]. As described in detail elsewhere, the number of megacapillaries is multiplied by the maximum diameter of the megacapillaries and then divided by the square of the number of capillaries to form the CSURI [8, 9]. For part of the analysis, we categorised the CSURI at 2.96, a threshold which was suggested to be predictive for the prospective development of DUs [9].

### Data analysis

Categorical variables were calculated as frequencies and percentages, and continuous variables were calculated as means with standard deviation (SD) or medians with interquartile range (IQR). Chi-square tests/Fisher’s exact tests and Mann–Whitney *U* tests were applied for across-group comparisons. Intraclass correlation coefficients and Cohen’s κ were calculated to assess the agreement between the two assessors. Linear regression analysis was applied to evaluate associations between the change in CSURI between baseline and follow-up and demographic or disease characteristics. All statistical analyses were performed with Stata/IC 13.1 (StataCorp., College Station, TX, USA).

## Results

Between 2011 and 2015, 61 patients from eight centres were enrolled. The median observation time was 1.0 year (IQR 1.0–1.1). Of these 61 patients, 24 patients according to central assessor 1 (39%) and 26 patients according to central assessor 2 (43%) had no megacapillaries present on any assessed finger either at baseline or at the follow-up visit (Table 2). Due to the absence of megacapillaries, the CSURI could not be calculated for those patients. Therefore, for only 34 of the 61 eligible patients (56%) was the CSURI scorable by both central assessors at both time points. This percentage of patients without megacapillaries was similar across all eight centres (*p* = 0.72).

**Table 2:** Overview of distribution of patients with absent megacapillaries at any of the assessed fingers (i.e. CSURI non-scorability) at baseline and follow-up according to the central assessors

	SSc patients (out of 61 patients) who had no megacapillaries on any of the assessed fingers		
	Central assessor 1	Central assessor 2	Both central assessors combined
Baseline	15 patients (25%)	17 patients (28%)	18 patients (30%)
Follow-up	15 patients (25%)	18 patients (30%)	18 patients (30%)
Any of the two time points	24 patients (39%)	26 patients (43%)	27 patients (44%)

*CSURI* capillaroscopic skin ulcer risk index, *SSc* systemic sclerosis

According to both central assessors, megacapillaries were present in 43 patients at baseline (Table 2); 30% of these showed an early SSc pattern on NC, 44% an active pattern and 26% a late pattern. Of the 18 patients without megacapillaries present at baseline (Table 2), 6% (one patient) had an early pattern, 28% a late pattern and the remaining 66% of patients showed no SSc specific pattern on NC at baseline.

The following analyses are entirely based on the 34 patients with an available CSURI by both central assessors at both time points, named the study population.

The baseline characteristics of the study population are presented in Table 3. The median observation time in this population was also 1.0 year (IQR 1.0–1.1). There were no statistically significant differences between the patients included in the study population and those excluded from further analysis. The included patients were, however, slightly younger (median age 57 years vs 62 years) and nominally more often had diffuse skin involvement (41% vs 33%) than the excluded patients. As many as 24% of the patients had experienced ulcer complications (soft tissue infections and gangrene).

**Table 3:** Comparison of baseline demographics and disease characteristics between patients included in this analysis (scorable CSURI at baseline and follow-up) and those excluded (CSURI not scorable at baseline and follow-up)

Baseline characteristic of study population	Included	Excluded	*p* value
*N*	34	27	
Age (years)	56.6 (47.8–64.8)	61.7 (53.6–64.6)	0.25
Male sex	26	30	0.79
Smoking habit			
Never smoker	47	37	0.23
Ex-smoker	18	37	
Current smoker	35	26	
Bosentan at any time during the observation period	38	33	0.69
Disease characteristics			
Time since RP onset (years)	7.0 (3–15)	5.0 (2–21)	0.65
Time since first non-RP manifestation (years)	4.5 (1–9)	5.0 (2–12)	0.44
Cutaneous involvement			
Limited	59	67	0.53
Diffuse	41	33	
Erectile dysfunction	13	44	0.29
Kidney involvement	0	4	0.45
History of renal crisis	0	0	–
RP condition score [20]	3.8 (2–7)	5.0 (2–7)	0.49
mRSS	8 (6–13)	9 (4–18)	0.63
Time since first DU (years)	1.5 (0.7–4.2)	2.3 (1.1–5.0)	0.42
DU	76	74	0.83
Number of DUs (in patients with DUs)	3.0 (1–7)	3.5 (1–6)	0.99
Previous major digital vascular complication			
None	76	69	0.39
Soft tissue infection	21	15	
Gangrene	3	8	
Autoamputation	0	8	
Laboratory parameters			
ANA positive	100	96	0.25
ACA positive	48	45	0.83
Scl-70 positive	34	45	0.46

Data presented as % or median (interquartile range)

*ACA* anticentromere autoantibodies, *ANA* anti-nuclear autoantibodies, *CSURI* capillaroscopic skin ulcer risk index, *DU* digital ulcer, *mRSS* modified Rodnan skin score, *RP* Raynaud’s phenomenon, *Scl-70* anti-topoisomerase I autoantibodies

In the study population, central assessor 1 counted a median of five capillaries in the distal row (range 2–10) and a median of one megacapillary (range 1–6) with a median maximum diameter of 62.5 μm (range 50–130 μm). Central assessor 2 counted a median of five capillaries in the distal row (range 2–10) and two megacapillaries (range 1–20) with a median diameter of 75 μm (range 30–180 μm).

The median baseline CSURI scores were 5.3 (IQR 2.6–16.3) as evaluated by central assessor 1 and 6.4 (IQR 2.4–12.5) as evaluated by central assessor 2. The median baseline CSURI was 8.2 (IQR 4.5–23.6) according to the local assessors. According to central assessor 1, the median CSURI score increased to 5.9 (IQR 1.3–12.0) at follow-up, whereas the median CSURI as evaluated by central assessor 2 decreased to 5.0 (IQR 1.7–10.0) at follow-up. The correlation coefficient between the baseline CSURI of the two assessors was 0.42, indicating a fair agreement [11]. There was a poor to fair agreement between the CSURI scored by the central assessors and the local assessors (central assessor 1/local assessors 0.45; central assessor 2/local assessors 0.38).

**Fig. 1:**
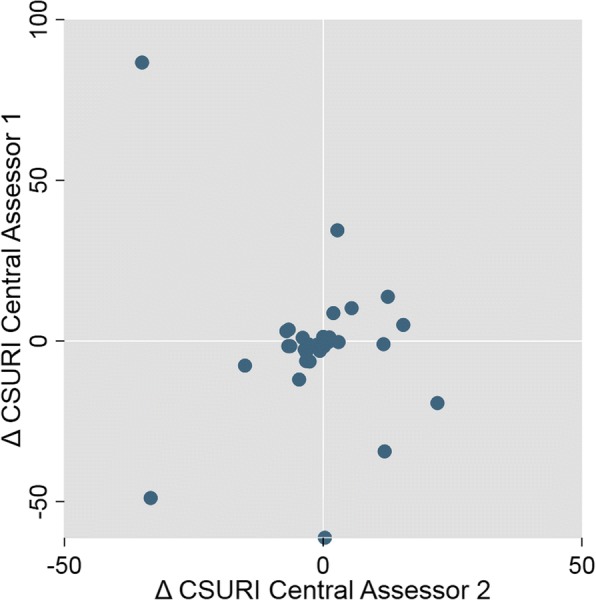
Change in CSURI between baseline and follow-up as evaluated by central assessors 1 and 2. CSURI capillaroscopic skin ulcer risk index

As evaluated by central assessor 1, 35% of patients had a higher CSURI at follow-up compared to 44% when evaluated by central assessor 2. In only 40% of the 34 patients was the change in CSURI between baseline and follow-up in the same direction for both central assessors; that is, an increase as measured by both assessors, a decrease in the measurements of both assessors or no change (Fig. 1).

According to both central assessors, 10 patients (29%) were in the low-risk category (CSURI < 2.96 [9]) at baseline; however, only seven of those 10 patients were concomitantly rated by both assessors as being in the low-risk category. According to the local assessors, five patients (15%) were in the low-risk category; however, only two of those were concomitantly rated by both central assessors into the low-risk category. The inter-assessor agreement of the central assessors for the baseline CSURI risk category was 0.58, indicating a moderate level of agreement [12]. The inter-assessor agreements of the local assessor and central assessor 1 or 2 were both 0.25, indicating a fair agreement.

Central assessor 1 scored 88% of the patients into the same risk category at baseline and at follow-up (i.e. either low–low risk or high–high risk); the remaining 12% were in the low-risk category at follow-up, but in the high-risk category at baseline. According to assessor 2, 73% of patients were in the same risk category at baseline and follow-up, 21% were in the high-risk category at baseline and in the low-risk category at follow-up, and 6% were in the low-risk category at baseline and in the high-risk category at follow-up. The agreement between the two central assessors of this ‘change in risk categories’ was fair (κ = 0.37) [12]. There was no agreement between central assessor 1 and central assessor 2 and the local assessors regarding this ‘change of risk category’ (κ = – 0.09, κ = – 0.16, respectively).

The prevalence of DU at baseline was 76% (Table 3) compared to 59% at follow-up. The ability of CSURI ≥ 2.96 (i.e. the high-risk category) to predict a higher number of DUs at follow-up than at baseline visit was rather low (positive predictive value for both central assessors 38%, for local assessors 48%), as was the ability of CSURI < 2.96 (i.e. low-risk category) to predict fewer or the same number of DUs at follow-up compared to baseline (negative predictive value for both central assessors 50%, for local assessors 67%). Out of the 34 included patients, 28 patients were classified into the same risk category by both central assessors. The positive and the negative predictive values based on these 28 patients were similarly lower (positive predictive value 38%, negative predictive value 43%) than the predictive values based on all 34 patients. The predictive values in patients who were treated with bosentan at any time during the observation period were similar to those who were not treated with bosentan.

No demographic or disease characteristic was associated with the change in the CSURI between baseline and follow-up simultaneously for both CSURIs, the one scored by assessor 1 and the one scored by assessor 2, in univariate linear regression (Table 4).

**Table 4:** Univariate linear regression of ΔCSURI (defined as the difference of CSURI between baseline and follow-up) and demographics and disease characteristics (*n* = 34)

Characteristic of study population	Central assessor 1			Central assessor 2		
	*β*	95% CI	*p* value	*β*	95% CI	*p* value
Age (years)	−0.095	−0.75 to 0.56	0.77	0.222	−0.08 to 0.53	0.15
Male sex	−0.92	−19.2 to 17.4	0.92	−3.5	−12.2 to 5.3	0.42
Smoking habit						
Never smoker	Reference			Reference		
Ex-smoker	3.61	−18.7 to 25.9	0.74	−1.4	− 12.3 to 9.5	0.79
Current smoker	11.54	−6.2 to 29.3	0.20	−3.9	−12.7 to 4.8	0.36
Disease characteristics						
Time since RP onset (years)	−0.093	−0.75 to 0.56	0.77	0.096	−0.19 to 0.39	0.50
Time since first non-RP manifestation (years)	−1.16	−2.9 to 0.6	0.18	0.91	0.1 to 1.7	0.025
Time since first DU (years)	−0.81	−3.0 to 1.4	0.46	0.90	0.0 to 1.8	0.046
Previous major digital vascular complication						
None	Reference			Reference		
Soft tissue infection	−5.62	−25.9 to 14.6	0.58	3.15	−6.4 to 12.7	0.51
Gangrene	5.24	−43.2 to 53.7	0.83	14.74	−8.1 to 37.5	0.20
Cutaneous involvement						
Limited	Reference			Reference		
Diffuse	4.05	−12.3 to 20.4	0.62	−3.08	−10.9 to 4.8	0.43
Erectile dysfunction	−7.99	−14.2 to −1.8	0.019	−7.28	−21.1 to 6.5	0.25
RP condition score [20] at baseline	−0.51	−2.4 to 1.4	0.59	−0.11	−1.0 to 0.8	0.81
mRSS at baseline	−0.04	−0.9 to 0.9	0.93	− 0.002	−0.4 to 0.4	0.99
Number of DUs at baseline	1.05	−0.8 to 2.9	0.25	0.55	−0.3 to 1.4	0.21
Laboratory parameters						
ACA positive	6.08	−10.4 to 22.5	0.46	−0.02	−8.1 to 8.0	0.99
Scl-70 positive	−16.5	−34.8 to 1.8	0.075	−2.73	−12.1 to 6.7	0.56

*ACA* anticentromere autoantibodies, *CI* confidence interval, *CSURI* capillaroscopic skin ulcer risk index, *DU* digital ulcer, *mRSS* modified Rodnan skin score, *RP* Raynaud’s phenomenon, *Scl-70* anti-topoisomerase autoantibodies

## Discussion

This prospective, longitudinal study examined the use of the CSURI in everyday clinical practice and demonstrates that 40% of patients in this multicentre study could not be evaluated with the CSURI at baseline and follow-up visits, mainly due to a normal NC pattern and the lack of any megacapillary as a prerequisite for the calculation of the CSURI [9, 13]. Additionally, the agreement of the CSURI between the two trained and experienced assessors was mediocre at best, as was the agreement between the two central assessors and the local assessors.

Our high percentage of non-scorable patients contrasts with the first CSURI study and the CSURI validation study [8, 9]. In the first study all patients had megacapillaries present, and in the second study only 13 out of an unselected SSc population of 242 patients (5%) were excluded from the study due to the absence of megacapillaries [8, 9]. However, in various other studies that were not applying the CSURI, the percentage of patients without megacapillaries was comparable to our high percentage. For instance, in a study of 188 SSc patients at least one quarter of patients had no megacapillaries [14]. Similarly, in two other studies, 24% and 30% of patients had no megacapillaries in any of the assessed fingers [15, 16]. Our discrepancies with the first CSURI studies are difficult to explain with differences of equipment, given the fact that very similar devices were in use in the first CSURI studies as well as in our study. Our patient population had similar disease duration as the patients consecutively recruited into the first CSURI study [8], but a higher proportion of diffuse SSc patients (41% vs 9%), which may not explain the lower prevalence of megacapillaries in our study.

In our study, the CSURI had only fair to moderate inter-rater reliability. This contrasts with an ‘almost perfect’ inter-observer reproducibility reported by Sebastiani et al. [8] in the original CSURI study, with κ = 0.96 based on the CSURI, dichotomised at the 2.96 cut-off value. A slightly lower but still ‘almost perfect’ inter-rater agreement of 0.85 was found in the validation study [9]. It is unlikely that these discrepancies can be completely explained with a lack of experience or different training, as both central assessors were trained together by authors of the original CSURI publications and used the same digital images and imaging software.

The CSURI was created as a prognostic index to predict the onset of new DUs [8]. In a validation study, Sebastiani et al. [9] demonstrated high predictive values for the development of DUs within 3 months, especially a high negative predictive value of 97%, but also a high positive predictive value of the CSURI of 81% in patients with a history of DUs. However, in another study by Sebastiani et al. [17] a poorer performance of the CSURI with lower predictive values was also observed in a population of SSc patients treated with bosentan. Differences in DU prediction may therefore be explained by differences in vasoactive medications. When we stratified our patients by bosentan treatment, we did not observe major differences in predictive values. It must, however, also be kept in mind that the predictive values from our study should not be directly compared with the studies by Sebastiani et al. as we assessed the predictive values of a higher number of DUs at follow-up compared to baseline and not ‘incident DU’ as Sebastiani et al. Additionally, the time between the baseline and the follow-up visit was considerably longer in our study (median time 1 year) than in Sebastiani et al.’s studies (3 months), which could also partly explain the differences in the predictive power of the CSURI.

A recent systematic literature review critically appraising studies reporting the prognostic value of NC in SSc also assessed the predictive value of the CSURI [18]. In line with our study, Paxton and Pauling [18] conclude that it is difficult to draw robust conclusions regarding the prognostic role of the CSURI; the reason for this being high levels of potential biases relating to study confounding as well as the statistical analyses.

It needs to be mentioned that our study has a rather limited sample size, which restricts the power to assess CSURI predictors in terms of demographic and disease characteristics. However, the mediocre performance of the CSURI regarding the inter-rater differences, as well as the high number of patients who could not be included due to the absence of megacapillaries, will not be a result of chance alone, even if a larger sample size would naturally have been beneficial.

## Conclusions

The CSURI was not applicable in a large percentage of patients due to the absence of megacapillaries and demonstrated only fair to moderate inter-rater reliability. Thus, in routine clinical practice, the CSURI should be used with caution for treatment decisions and prediction of incident DUs.

## Additional file

Additional file 1: **Table S1.** Inclusion and exclusion criteria. (DOCX 16 kb)

## Abbreviations

CSURI: Capillaroscopic skin ulcer risk index
DU: Digital ulcer
IQR: Interquartile range
NC: Nailfold capillaroscopy
RP: Raynaud’s phenomenon
SD: Standard deviation
SSc: Systemic sclerosis
