# Supplementary material for: Prospective evaluation of the capillaroscopic skin ulcer risk index in systemic sclerosis patients in clinical practice: a longitudinal, multicentre study
Source: Arthritis Res Ther. 2018 Oct 25;20:239. doi: 10.1186/s13075-018-1733-6 (PMC6235233; doi:10.1186/s13075-018-1733-6)
Supplement: Supplementary file 1 — Table S1. Inclusion and exclusion criteria. (DOCX 16 kb) [file 13075_2018_1733_MOESM1_ESM.docx]

**Prospective Evaluation of the Capillaroscopic Skin Ulcer Risk Index (CSURI) in Clinical Practice**

Ulrich A. Walker, Veronika K. Jaeger, Katharina M. Bruppacher, Rucsandra Dobrota, Lionel Arlettaz, Martin Banyai, Jörg Beron, Carlo Chizzolini, Ernst Groechenig, Rüdiger B. Mueller, François Spertini, Peter M. Villiger, Oliver Distler

**Table S1** Inclusion and exclusion criteria.

| ***Inclusion criteria*** |
| --- |
| Definite diagnosis of systemic sclerosis according to the 1980 ACR criteria |
| Digital ulcer at enrollment or history of digital ischemic ulcers secondary to systemic sclerosis within the past year prior to enrollment |
| Signed patient informed consent form |
| ***Exclusion criteria*** |
| Digital ulcers due to a condition other than systemic sclerosis |
| Digital ulcers associated with calcinosis |
| Treatment with parenteral prostanoids or bosentan within the past 3 months before enrollment |
| Treatment with phosphodiesterase type 5 inhibitors, except for intermittent treatment of erectile dysfunction |
| Treatment with other endothelin receptor antagonist |
| Any other experimental treatment |
| Systemic antibiotics for infected ulcers |
| Active renal crisis or advanced cardiopulmonary disease or otherwise clinically unstable patients |
